# Impact of polypharmacy on 3-year mortality in patients with heart failure: a retrospective study

**DOI:** 10.1186/s40780-024-00357-7

**Published:** 2024-07-02

**Authors:** Daisuke Hayashi, Yoshiaki Kubota, Takuya Nishino, Yukihiro Watanabe, Yoshiki Iwade, Junya Matsuda, Katsuhito Kato, Shuhei Tara, Yuya Ise, Yu-ki Iwasaki, Kuniya Asai

**Affiliations:** 1 Department of Pharmaceutical Service, Nippon Medical School Hospital, 1-1-5 Sendagi, Bunkyo-Ku, Tokyo, 113-8603 Japan; 2 Department of Cardiovascular Medicine, Nippon Medical School, 1-1-5 Sendagi, Bunkyo-Ku, Tokyo, 113-8603 Japan; 3 Department of Health Care Administration, Nippon Medical School, 1-1-5 Sendagi, Bunkyo-Ku, Tokyo, 113-8603 Japan; 4 Department of Hygiene and Public Health, Nippon Medical School, 1-1-5 Sendagi, Bunkyo-Ku, Tokyo, 113-8603 Japan

**Keywords:** Polypharmacy, Guideline-directed medical therapy, Heart failure, Drug count

## Abstract

**Background:**

Guideline-directed medical therapy (GDMT) is important in heart failure management; however, polypharmacy itself may impact heart failure. Although measures against polypharmacy are needed, current discussion on unilateral drug tapering (including the drugs that should be tapered) is insufficient. In this study, we investigated the relationship between the number of prescribed GDMT drugs and prognosis in patients with heart failure.

**Methods:**

In this single-centre retrospective study, 3,146 eligible patients with heart failure were included and divided into four groups based on the median number of prescribed GDMT drugs and the median number of drugs not included in the GDMT (ni-GDMT) at the time of hospital discharge. The definition of GDMT was based on various Japanese guidelines. The primary outcome was all-cause mortality within 3 years of hospital discharge.

**Results:**

A total of 252 deaths were observed during the 3-year follow-up period. Kaplan–Meier analysis revealed that groups with GDMT drug count ≥ 5 and ni-GDMT drug count < 4 had the lowest mortality, and those with GDMT drug count < 5 and ni-GDMT drug count ≥ 4 had the highest mortality (log-rank, *P* < 0.001). Cox regression analysis revealed a significant association between ni-GDMT drug count and all-cause mortality, even after adjustment for number of GDMT medications, age, male, left ventricular ejection function < 40%, hemoglobin, albumin levels, and estimated glomerular filtration rate [HR = 1.06 (95% CI: 1.01–1.11), *P* = 0.020]. Conversely, the GDMT drug count was not associated with increased mortality rates.

**Conclusions:**

The ni-GDMT drug count was significantly associated with 3-year mortality in patients with heart failure. Conversely, the GDMT drug count did not worsen the prognosis. Polypharmacy measures should consider ni-GDMT drug quantity to improve the prognosis and outcomes in patients with heart failure.

**Supplementary Information:**

The online version contains supplementary material available at 10.1186/s40780-024-00357-7.

## Background

Polypharmacy is a century-old term. Currently, it is defined as the use of five or more drugs [1]. This cut-off point is associated with the risk of adverse outcomes in older adults, such as falls, frailty, disability, and death [1]. Guideline-directed medical therapy (GDMT) is important in heart failure treatment [2, 3]. Angiotensin receptor-neprilysin inhibitors (ARNIs), beta blockers (BBs), mineralocorticoid receptor antagonists (MRAs), and sodium-glucose co-transporter-2 inhibitors (SGLT2i) are recommended medications for patients with heart failure with a decreased ejection fraction [4]. Strengthening GDMT increases the risk of polypharmacy occurrence. Polypharmacy use is more common among patients with heart diseases [5, 6]. Although polypharmacy itself may increase the risk of all-cause hospitalisation, it reportedly has no effect on mortality [7]. Antiarrhythmics, calcium channel blockers, nonsteroidal anti-inflammatory drugs, and thiazolidinediones are listed in the American College of Cardiology/American Heart Association guidelines as drugs that cause adverse outcomes in patients with heart failure [8]. A small-scale review of drug therapy tapering in patients with heart failure revealed that the discontinuation of renin-angiotensin aldosterone system inhibitors and BBs had an adverse impact on key outcomes [9]. Despite the importance of polypharmacy measures in patients with heart failure, sufficient discussions on the unilateral implementation of drug tapering (regardless of the drug type), optimal drug therapy, and the drug’s effect on prognosis are currently lacking. Therefore, we aimed to examine the impact of polypharmacy on the prognosis of patients with heart failure.

## Methods

### Research design and data collection

This single-centre retrospective cohort study was based on our inpatient database. Data including the patients’ age, sex, diagnosis and comorbidities, treatment modalities, medication history, results of various tests, and outcomes after hospital discharge were extracted from the medical records. The diagnostic definitions were based on the 10th edition of the International Classification of Diseases (Additional file 1). This study conforms to the principles outlined in the 1964 Declaration of Helsinki and its later amendments and was approved by the ethics committee of the Nippon Medical School Hospital (Tokyo, Japan, B-2021–433). Participants were provided with an explanation of the study, and their consent was obtained using an opt-out form.

### Participant selection

**Fig. 1:**
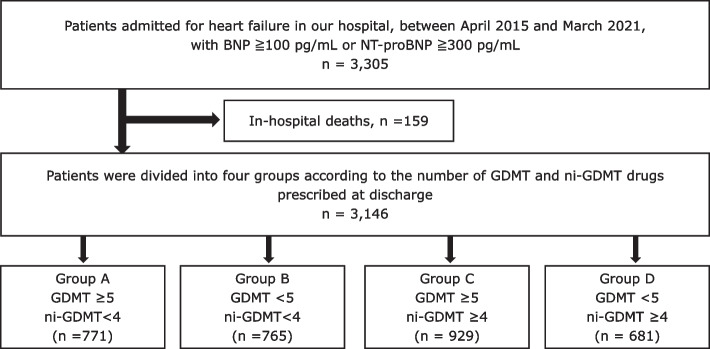
Flow chart. GDMT, Guideline-directed medical therapy; ni-GDMT, not included in the guideline-directed medical therapy; BNP, brain natriuretic peptide; NT-proBNP, N-terminal prohormone of brain natriuretic peptide

Patients with heart failure (defined as having a brain natriuretic peptide [BNP] level of ≥ 100 pg/mL or an N-terminal pro-brain natriuretic peptide [NT-proBNP] level of ≥ 300 pg/mL) who were admitted to our hospital between April 2015 and March 2021 were considered for inclusion in this study. After excluding in-hospital deaths, 3,146 patients were finally included, and their data were extracted for analysis (Fig. 1). The point in time of the New York Heart Association (NYHA), laboratory and echocardiography data were at 48-h and 1-week before discharge, respectively.

### Definitions of GDMT and polypharmacy

GDMT was defined according to various Japanese guidelines [10–13] (Additional file 2); ‘not included in the GDMT’ (i.e., ni-GDMT) comprised all other prescribed drugs (Additional file 3). Antiplatelet agents and anticoagulants were defined as GDMT because they are more likely to be associated with ischemic heart disease and atrial fibrillation, which are direct causes of heart failure and are therefore considered relevant in the treatment of heart failure, and proton pump inhibitors (PPIs) are the drugs recommended in the cited guideline algorithm and are more strongly recommended than histamine type-2-receptor antagonist (H_2_ RA) [13]. H_2_ RA was defined as ni-GDMT because it is classified as potentially inappropriate, which can be an inappropriate drug, especially in elderly patients. For polypharmacy, the median GDMT and ni-GDMT drug counts of all the participants were calculated; based on these values, the participants were divided into four groups (Additional file 4): Group A (GDMT drug count ≥ 5, ni-GDMT drug count < 4), Group B (GDMT drug count < 5, ni-GDMT drug count < 4), Group C (GDMT drug count ≥ 5, ni-GDMT drug count ≥ 4), and Group D (GDMT drug count < 5, ni-GDMT ≥ 4).

### Outcomes

The primary endpoint was all-cause mortality within 3 years of hospital discharge. The secondary endpoints were cardiac and non-cardiac deaths within 3 years of hospital discharge. Cardiac death was defined as death due to cardiogenic shock, heart failure, arrhythmia, or myocardial infarction; non-cardiac death was defined as death other than cardiac death.

### Statistical analysis

Categorical variables are expressed as numbers and percentages; these were compared among the groups using the chi-square test. Continuous variables are expressed as means and standard deviations or as medians and interquartile ranges (IQRs).

The 3-year all-cause mortality rate was analysed using the Kaplan–Meier method, and intergroup differences were determined using a log-rank test. To compare the cumulative incidence of competing risks, the Gray's test was used, defining competing risks in the data set as cardiac or noncardiac deaths. This test was designed to handle multiple types of events so that the presence of competing risks would not bias the results. A cumulative incidence function was estimated for each event to determine whether there was a statistically significant difference between groups [14]. A Cox regression analysis was performed to identify the independent factors associated with all-cause mortality. For multivariable analysis, the following variables were considered: number of GDMT medications, number of ni-GDMT medications, age, LVEF < 40%, haemoglobin and albumin levels, and estimated glomerular filtration rate (eGFR). For sub-group analyses, the effects of age, sex, ischemic heart disease (IHD), diabetes, and left ventricular ejection fraction (LVEF) on all-cause mortality were investigated. Statistical analysis was performed using R ver. 4.2.1 (R Foundation for Statistical Computing, Vienna, Austria). A two-sided *P* value < 0.05 was considered statistically significant.

## Results

### Baseline characteristics

Among the 3,146 participants included, 771, 765, 929, and 681 were categorised into Groups A, B, C, and D, respectively. The median drug counts were 8 (IQR: 7–9), 4 (IQR: 2–6), 12 (IQR: 11–14), and 9 (IQR: 7–10) in Groups A, B, C, and D, respectively (Table 1).

**Table 1:** Characteristics stratified by the number of GDMT and ni-GDMT drugs in patients with heart failure

Variables	A GDMT ≥5 ni-GDMT <4 (*n* = 771)	B GDMT <5 ni-GDMT <4 (*n* = 765)	C GDMT ≥5 ni-GDMT ≥4 (*n* = 929)	D GDMT <5 ni-GDMT ≥4 (*n* = 681)
Demographics				
Age (years)	72.2 (13.0)	73.6 (15.6)	76.5 (11.4)	77.9 (12.0)
Male sex, n (%)	515 (66.8)	426 (55.7)	567 (61.0)	61 (53.0)
Bedridden or Wheelchair	41 (5.3)	110 (14.4)	87 (9.4)	103 (15.1)
Number of medicines				
All	8 (7, 9)	4 (2, 6)	12 (11, 14)	9 (7, 10)
GDMT	6 (5, 7)	3 (1, 4)	6 (5, 7)	3 (2, 4)
ni-GDMT	2 (1, 3)	2 (1, 3)	6 (4, 7)	6 (4, 7)
Symptoms				
NYHA functional classification				
I, n (%)	51/170 (29.8)	62/140 (44.3)	68/259 (26.3)	48/145 (33.1)
II, n (%)	57/170 (33.3)	37/140 (26.4)	98/259 (37.8)	46/145 (31.7)
III, n (%)	42/170 (24.6)	25/140 (17.9)	56/259 (21.6)	33/145 (22.8)
IV, n (%)	21/170 (12.3)	16/140 (11.4)	37/259 (14.3)	18/145 (12.4)
Comorbidities				
Hypertension, n (%)	663 (86.0)	565 (73.9)	773 (83.2)	510 (74.9)
Dyslipidaemia, n (%)	545 (70.7)	292 (38.2)	536 (57.7)	259 (38.0)
Diabetes mellitus, n (%)	265 (34.4)	159 (20.8)	406 (43.7)	193 (28.3)
Acute coronary syndrome, n (%)	300 (38.9)	84 (11.0)	207 (22.3)	64 (9.4)
Ischaemic heart disease, n (%)	450 (58.4)	141 (18.4)	433 (46.6)	115 (16.9)
Echocardiography				
LVEF, n (%)	48.9 (18.1)	57.1 (17.4)	48.0 (18.4)	58.1 (16.4)
Laboratory data at discharge				
Haemoglobin (g/dL)	12.6 (2.1)	12.2 (2.2)	11.4 (2.0)	11.1 (1.8)
Albumin (g/dL)	3.5 (0.5)	3.4 (0.6)	3.4 (0.5)	3.2 (0.6)
eGFR (mL/min/1.73 m^2^)	55.1 (20.2)	60.1 (24.0)	45.6 (24.3)	50.5 (29.6)
Uric acid (mg/dL)	6.3 (1.8)	6.3 (1.8)	6.2 (2.0)	5.6 (2.0)
NT-proBNP (pg/mL)	2951(1220, 7171)	2232(953, 5789)	4130 (1901, 9564)	2364 (936, 7187)
Medications at discharge				
Beta blocker, n (%)	669 (86.8)	297 (45.0)	773 (83.3)	289 (44.1)
ACE-I, n (%)	445 (57.7)	139 (21.1)	380 (40.9)	96 (14.6)
ARB, n (%)	251 (32.6)	166 (25.2)	397 (42.8)	218 (33.2)
MRA, n (%)	395 (51.2)	124 (18.8)	466 (50.2)	95 (14.5)
SGLT2i, n (%)	51 (6.6)	10 (1.3)	74 (8.0)	7 (1.0)
Aspirin, n (%)	434 (56.3)	105 (15.9)	413 (44.5)	123 (18.8)
Thienopyridine, n (%)	389 (50.5)	65 (9.8)	380 (40.9)	68 (10.4)
Warfarin, n (%)	101 (13.1)	28 (4.2)	190 (20.5)	42 (6.4)
Direct oral anticoagulant, n (%)	258 (33.5)	237 (35.9)	315 (33.9)	165 (25.2)
Loop diuretic, n (%)	407 (52.8)	190 (28.8)	639 (68.9)	210 (32.0)
Tolvaptan, n (%)	74 (9.6)	12 (1.8)	197 (21.2)	35 (5.3)
Statin, n (%)	555 (72.0)	175 (26.5)	625 (67.3)	188 (28.7)
Proton pump inhibitor, n (%)	617 (80.0)	293 (44.4)	754 (81.2)	349 (53.2)

Continuous data are presented as mean values (standard deviation) or median values (interquartile ranges). Categorical data are presented as n (percentages)

*GDMT* Guideline-directed medical therapy, *ni-GDMT* not included in the Guideline-directed medical therapy, *ACE-I* angiotensin-converting enzyme inhibitors, *ARB* angiotensin-receptor blockers, *MRA* mineralocorticoid receptor antagonists, *SGLT2i* Sodium-glucose cotransporter 2 inhibitors, *eGFR* estimated glomerular filtration rate, *LVEF* left ventricular ejection fraction, *NT-proBNP* N-terminal prohormone brain natriuretic peptide

### Primary endpoint

**Fig. 2:**
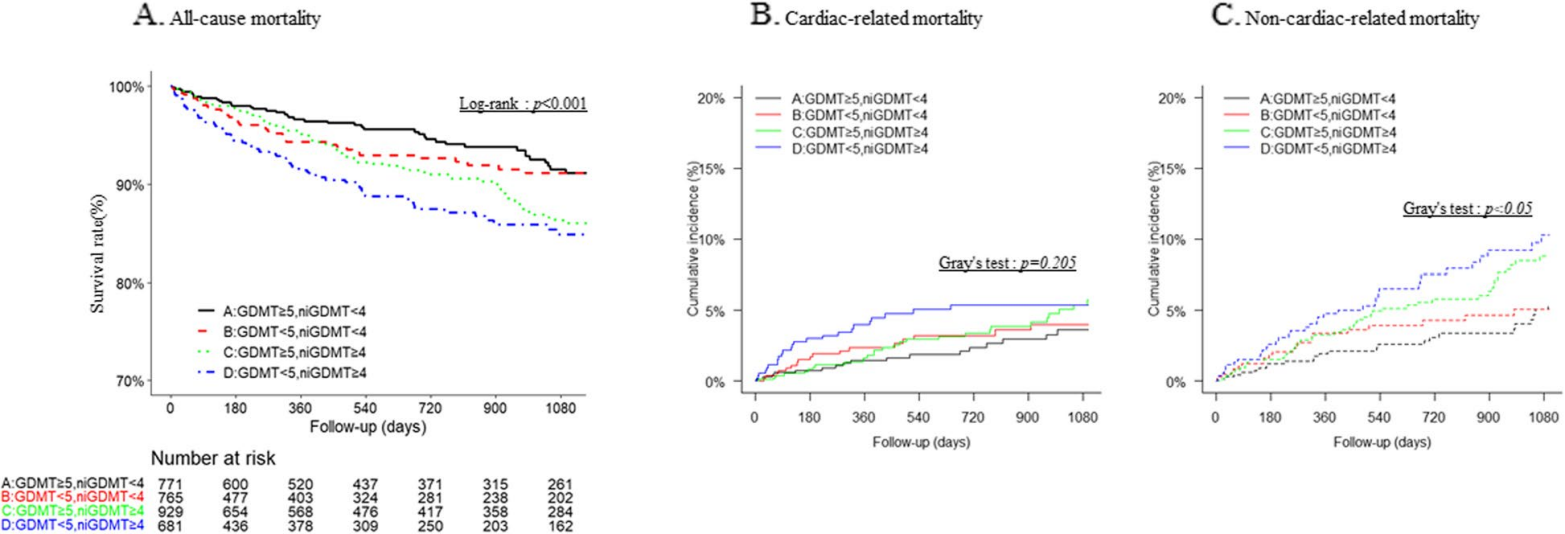
Primary and secondary endpoints by number of GDMT and ni-GDMT drugs. Primary endpoint: Kaplan-Meier curve for all-cause mortality (**A**); secondary endpoints: cumulative incidence of cardiac death due to shock, heart failure, arrhythmia, and myocardial infarction (**B**); cumulative incidence of other noncardiac death (**C**)

Figure 2A illustrates the 3-year all-cause mortality rate. A total of 252 deaths were observed during the 3-year follow-up period. Kaplan–Meier analysis revealed that groups with GDMT drug count ≥ 5 and ni-GDMT drug count < 4 had the lowest mortality and those with GDMT drug count < 5 and ni-GDMT drug count ≥ 4 had the highest mortality. The mortality rates were 3.4%, 5.7%, 4.9%, and 8.5% in Groups A, B, C, and D, respectively; these differences were significant (Log-rank *p* < 0.001). When the competing risks from cardiac and noncardiac deaths were taken into account in the comparison, there was no significant difference between the groups for cardiac deaths. On the other hand, the cumulative incidence of non-cardiac death was higher in group D and significantly lower in group A (Gray's test < 0.05).

### Adjusted 3-year mortality rate

Table 2 presents the 3-year mortality rate analysed using Cox proportional hazards models. Compared with Group A, Group C and D had a significantly increased mortality rate [HR = 1.64 (95% CI: 1.12–2.41), *P* = 0.007), HR = 2.08 (95% CI: 1.40–3.09), *P* < 0.001].

**Table 2:** Cox regression analysis for all-cause mortality within 3 years

Group	HR (95% CI)
A. GDMT ≥5, ni-GDMT <4	ref.
B. GDMT <5, ni-GDMT <4	1.21 (0.78-1.88)
C. GDMT ≥5, ni-GDMT ≥4	1.64* (1.12-2.41)
D. GDMT <5, ni-GDMT ≥4	2.08* (1.40-3.09)

*HR* hazard ratio, *CI* confidence interval, *GDMT* Guideline-directed medical therapy, *ni-GDMT* not included in the Guideline-directed medical therapy

*P<0.05

### Sub-group analyses in ni-GDMT

**Fig. 3:**
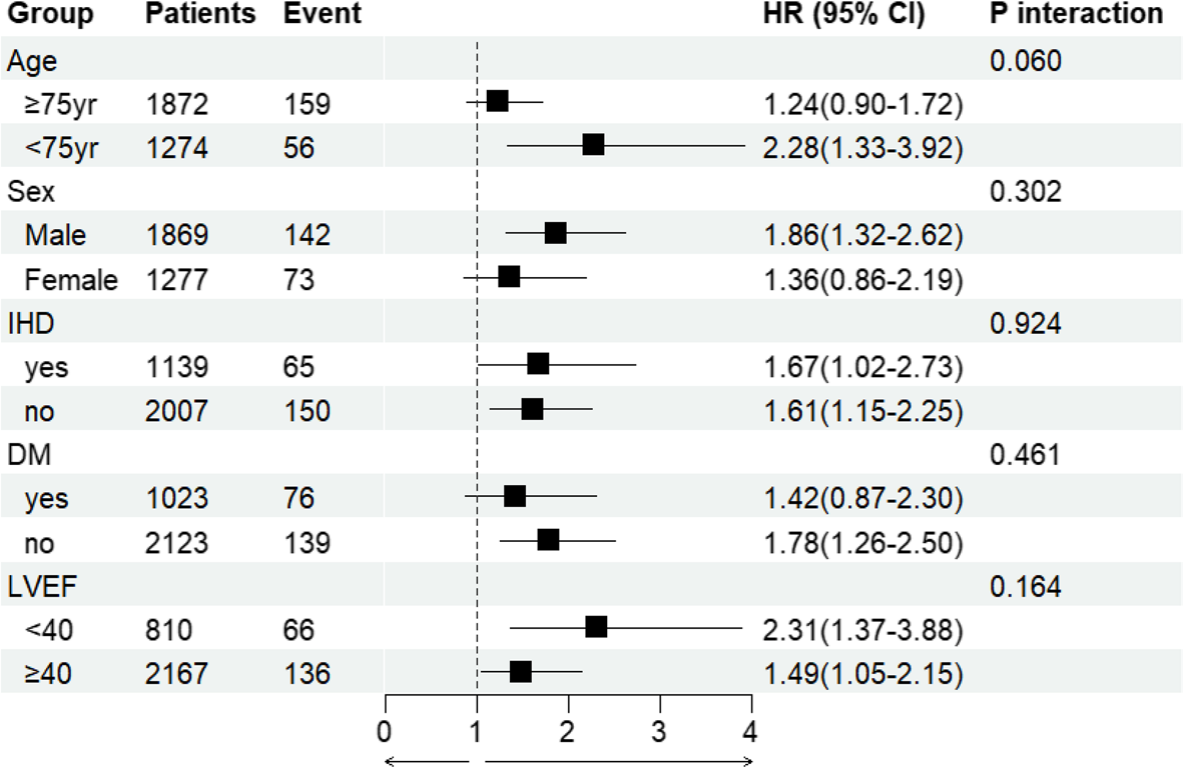
Sub-group analysis of the number of ni-GDMT drugs and 3-year mortality. Association between the ni-GDMT drug count and 3-year mortality rate examined using sub-group analyses; the variables were age ≥75 or <75 years, sex, presence or absence of IHD, presence or absence of diabetes, and left ventricular ejection fraction ≥40% or <40%. ni-GDMT, not included in the guideline-directed medical therapy; IHD, Ischemic Heart Disease; LVEF, left ventricular ejection fraction; DM, diabetes mellitus; CI, confidence interval; HR, hazard ratio

In the subgroup analysis (Fig. 3), no interaction was observed for age < 75 years, male, IHD, diabetes mellitus, or LVEF < 40%.

### Cox regression analysis of all-cause mortality within 3 years

Cox regression analysis showed a significant association between the number of ni-GDMT drugs and all-cause mortality, even after adjustment for number of GDMT medications, age, male, LVEF < 40%, hemoglobin, albumin levels, and eGFR [HR = 1.06 (95% CI: 1.01–1.11), *P* = 0.020; Table 3].

**Table 3:** Multivariable analysis of all-cause mortality within 3 years

Factor	HR	95% CI	*P*
Number of ni-GDMT drugs	1.06	1.01-1.11	0.020
Number of GDMT drugs	0.94	0.88-1.00	0.066
Other factors			
Age (years)	1.05	1.03-1.06	<0.001
Male sex	1.71	1.26-2.32	<0.001
>LVEF <40%	1.73	1.27-2.35	<0.001
Haemoglobin (g/dL)	0.82	0.74-0.90	<0.001
Albumin (g/dL)	0.42	0.31-0.57	<0.001
eGFR (mL/min/1.73 m^2^)	0.99	0.99-1.00	0.029

*HR* hazard ratio, *CI* confidence interval, *GDMT* Guideline-directed medical therapy, *ni-GDMT* not included in the Guideline-directed medical therapy, *eGFR* estimated glomerular filtration rate, *LVEF* left ventricular ejection fraction

Cox regression multivariable analysis included number of GDMT medications, number of ni-GDMT medications, age, male, LVEF <40%, haemoglobin, albumin levels, and eGFR level

### Cardiac- and non-cardiac-related deaths within 3 years

The mortality rates in each group with all deaths were divided into cardiac- and non-cardiac-related deaths. Regarding cardiac-related deaths, the mortality rates did not differ significantly among the four groups. However, regarding non-cardiac-related deaths, the mortality rates differed among all the groups (Table 4). For both cardiac- and non-cardiac-related deaths, the GDMT drug count was not associated with an increased mortality rate; however, for non-cardiac-related deaths, the ni-GDMT drug count was associated with an increased mortality rate.

**Table 4:** Secondary endpoints; comparison of cardiac- and non-cardiac-related deaths within 3 years in each group

Variables	A GDMT ≥ 5 ni-GDMT < 4 (*n* = 771)	B GDMT < 5 ni-GDMT < 4 (*n* = 765)	C GDMT ≥ 5 ni-GDMT ≥ 4 (*n* = 929)	D GDMT < 5 ni-GDMT ≥ 4 (*n* = 681)	*P* value
Cardiac-related death	17 (2.2)	17 (2.2)	28 (3.0)	24 (3.5)	0.329
Non-cardiac-related death	24 (3.1)	22 (2.9)	46 (5.0)	37 (5.4)	0.022

Data are reported as number (percentages). *P* values were obtained by chi-square-test with significance set at *P* < 0.05

*GDMT* Guideline-directed medical therapy, *ni-GDMT* not included in the Guideline-directed medical therapy

## Discussion

We investigated the relationship between the GDMT and ni-GDMT drug counts and the 3-year mortality rate in patients with heart failure. Our results revealed that the mortality rate within 3 years of hospital discharge was the lowest in groups with a high GDMT drug count and low ni-GDMT drug count. The GDMT drug count was not associated with an increased 3-year mortality; however, the ni-GDMT drug count had an increased 3-year mortality rate, particularly non-cardiac-related deaths. To the best of our knowledge, this study is the first to clarify that while ni-GDMT polypharmacy influenced the mortality rates, an increase in the GDMT drug count did not increase the mortality rates.

The definition of polypharmacy remains unclear; however, a review revealed that in 46.4% of 110 reports published between 1 January 2000 and 30 May 2016, polypharmacy was defined as the use of five or more daily medications [15]. Additionally, in some reports, hyper-polypharmacy was defined as the use of ≥ 10 drugs [7]. Reportedly, polypharmacy and heart disease may increase the risk of falls among older adults [16]. It may also increase the risk of death, particularly among older adults and patients with frailty [17, 18]. These reports suggest that polypharmacy itself can adversely affect health [19].

Polypharmacy was reported to have increased the re-hospitalisation rate due to adverse events, though not the mortality rate, in patients with heart failure with a preserved ejection fraction [20].

The drug counts that were considered in the present study were 8, 4, 12, and 9 in Groups A, B, C, and D, respectively; all groups, except Group B, satisfied the definition of polypharmacy. From the perspective of polypharmacy, Groups B and C were assumed to have the lowest and highest mortality risks, respectively; however, the results revealed that the mortality rate decreased in the order of Groups D > B > C > A. This indicates that simply increasing the number of drugs does not increase the risk of mortality. The 3-year mortality rate was observed to increase significantly with a higher ni-GDMT drug count. In a Canadian population-based study, approximately 20% of the study population took GDMT drugs (such as ACE-I/ARB or ARNI, BB, and MRA) [21]. A recent report indicated high mortality among patients who were unable to sufficiently initiate GDMT [4]. Additionally, recent reports have also indicated that prompt introduction of GDMT by up-titration in heart failure treatment may help ameliorate heart failure symptoms, improve quality of life, and reduce the risk of re-hospitalisation and mortality [22]. Although guideline recommendations vary between patients with maintained and decreased ejection fraction, it has recently been shown that GDMT may be beneficial even in patients with a maintained ejection fraction [23].

A report examining the incidence of gastrointestinal bleeding (GIB) and its subsequent impact on prognosis in patients with chronic heart failure reported that patients who experienced GIB had significantly higher rates of cardiac events and all-cause mortality compared to those who did not experience GIB. Gastrointestinal bleeding is a significant prognostic factor in heart failure patients, especially in patients receiving antiplatelet therapy or anticoagulation therapy, and thus risk management and prevention of gastrointestinal bleeding is important [24]. In addition, prompt treatment and appropriate risk management of gastrointestinal bleeding in patients on anticoagulation are essential, with the concomitant use of PPIs recommended [25].

Subgroup analysis in this study showed no significant effect on 3-year mortality in the age < 75 years, male, IHD, diabetes and LVEF < 40% groups. This suggests that ni-GDMT polypharmacy for patients with heart failure in either group should be considered in clinical practice. Generally, inappropriate polypharmacy leads to adverse drug events, hospitalisations, and death. The number of prescribed drugs is an important predictor associated with the risk of inappropriate prescription and adverse drug events [26]. Therefore, drug tapering or discontinuation must be considered to minimise polypharmacy and improve patient outcomes [26]. In practice, patient care provided by heart failure teams is associated with the improvement of drug-related clinical outcomes and decreased hospitalisation and is effective from the perspective of health economics [27]. On the other hand, according to the Centres for Disease Control and Prevention, polypharmacy increases health risks such as falls, hospitalisations, and death, but the quality of evidence supporting this is low, with few reports of its effectiveness in preventing death, hospitalisations, and falls [28], and drug reduction strategies against polypharmacy. No convincing evidence exists on its effectiveness or that polypharmacy affects clinically relevant endpoints. Therefore, more effective strategies to reduce inappropriate polypharmacy should be developed [29].

Patients with heart failure have several comorbidities resulting in polypharmacy. The results of the present study suggest that polypharmacy should be considered from the perspectives of ni-GDMT drug quantity. Clinical pharmacists and physicians should collaborate when considering polypharmacy interventions in patients with heart failure. Complementary ‘quality-based’ polypharmacy measures for ‘quantity-based’ polypharmacy would involve prioritising drugs that impact the patient’s prognosis or quality of life.

### Limitations

This study has several limitations. First, this was a single-centre study with a small sample size. Second, data on ARNIs, SGLT2i and ivabradine were unavailable because they were not prescribed in our hospital during the study period. Third, detailed information on the aetiology of heart failure, NYHA classification or the number of comorbidities could not be obtained. A group of patients with high ni-GDMT may have multiple non-cardiac complications and low GDMT. Fourth, the definition of GDMT includes antiplatelet therapy (aspirin and thienopyridine), warfarin, and PPIs. However, guideline recommendations differ for patients with preserved and reduced ejection fraction. This study performed crude analyses that included all cardioprotective agents. Fifth, patients were divided into four groups based on the prescription at hospital discharge; hence, information on the post-discharge prescription was unavailable. Therefore, future multi-centre studies are needed to further verify the impact of polypharmacy on patients with heart failure.

## Conclusion

Polypharmacy with a high ni-GDMT drug count influenced the prognosis of patients with heart failure. Conversely, the GDMT drug count did not adversely affect the prognosis of these patients. Polypharmacy measures that focus on ni-GDMT drug quantity and collaboration between cardiologists and clinical pharmacists are important.

### Supplementary Information

Additional file 1. International Classification of Diseases, 10th Revision (ICD-10).

Additional file 2. Drugs included in guideline-directed medical therapy.

Additional file 3. ni-GDMT Drug Classification Percentage used.

Additional file 4. Patients were divided into four groups according to the number of GDMT and ni-GDMT drugs prescribed at discharge.

## Data Availability

The deidentified participant data will not be shared because the patients’ consent has not been obtained.

## Abbreviations

GDMT: Guideline-directed medical therapy
ARNI: Angiotensin receptor-neprilysin inhibitors
BB: Beta blockers
MRA: Mineralocorticoid receptor antagonists
SGLT2i: Sodium-glucose co-transporter-2 inhibitors
BNP: Brain natriuretic peptide
NT-proBNP: N-terminal pro-brain natriuretic peptide
ni-GDMT: Not included in the GDMT
IQR: Interquartile range
eGFR: Estimated glomerular filtration rate
IHD: Ischemic heart disease
LVEF: Left ventricular ejaculation fraction
HR: Hazard ratio
CI: Confidence interval
PPI: Proton pump inhibitors
H_2_ RA: Histamine type-2-receptor antagonists
NYHA: New York Heart Association
ACE-I: Angiotensin-converting enzyme inhibitors
ARB: Angiotensin-receptor blockers
GIB: Gastrointestinal bleeding
