# Supplementary material for: Impact of polypharmacy on 3-year mortality in patients with heart failure: a retrospective study
Source: J Pharm Health Care Sci. 2024 Jul 2;10:34. doi: 10.1186/s40780-024-00357-7 (PMC11221177; doi:10.1186/s40780-024-00357-7)
Supplement: Supplementary file 1 — Additional file 1. International Classification of Diseases, 10th Revision (ICD-10). [file 40780_2024_357_MOESM1_ESM.docx]

| Online Resource 1. International Classification of Diseases, 10th Revision (ICD-10) | |
| --- | --- |
| Heart failure | I50 |
| Hypertension | I10, I11, I12, I13, I15 |
| Dyslipidemia | E78 |
| Diabetes mellitus | E10, E11, E12, E13, E14 |
| Ischemic heart disease | I21, I22, I252, I255 |
