# Supplementary material for: Impact of polypharmacy on 3-year mortality in patients with heart failure: a retrospective study
Source: J Pharm Health Care Sci. 2024 Jul 2;10:34. doi: 10.1186/s40780-024-00357-7 (PMC11221177; doi:10.1186/s40780-024-00357-7)
Supplement: Supplementary file 2 — Additional file 2. Drugs included in guideline-directed medical therapy. [file 40780_2024_357_MOESM2_ESM.docx]

| Online Resource 2. Drugs included in guideline-directed medical therapy |
| --- |
| Beta-blocker |
| ACE-I / ARB |
| MRA |
| Antiplatelet agent (Aspirin and Thienopyridine) |
| Anticoagulant agent (Warfarin and Direct oral anticoagulant) |
| Diuretic (loop and Tolvaptan) |
| Statin |
| Proton pump inhibitor |

ACE-I, angiotensin-converting enzyme-inhibitor; ARB, angiotensin-receptor blocker; MRA, mineralocorticoid receptor antagonist
