# Supplementary material for: Impact of polypharmacy on 3-year mortality in patients with heart failure: a retrospective study
Source: J Pharm Health Care Sci. 2024 Jul 2;10:34. doi: 10.1186/s40780-024-00357-7 (PMC11221177; doi:10.1186/s40780-024-00357-7)
Supplement: Supplementary file 3 — Additional file 3. ni-GDMT Drug Classification Percentage used. [file 40780_2024_357_MOESM3_ESM.docx]

Online Resource 3. ni-GDMT Drug Classification Percentage used

| Ni-GDMT Drug Classification | Prescription ,n(%) |
| --- | --- |
| Gastrointestinal agents | 1613 (51.3) |
| Coronary vasodilator agents | 1177 (37.4) |
| Anti-hyperuricemia agents | 826 (26.3) |
| Anti-insomnia agents | 807 (25.7) |
| Oral hypoglycemic agents | 675 (21.5) |
| Antiarrhythmic drugs | 410 (13.0) |
| Antipsychotic drugs | 295 (9.4) |
| Urological drugs and Anorectal drugs | 260 (8.3) |
| Expectorant agents | 216 (6.9) |
| Antihypertensive drugs | 187 (5.9) |

ni-GDMT, not included in the Guideline-directed medical therapy
