# Supplementary material for: Impact of polypharmacy on 3-year mortality in patients with heart failure: a retrospective study
Source: J Pharm Health Care Sci. 2024 Jul 2;10:34. doi: 10.1186/s40780-024-00357-7 (PMC11221177; doi:10.1186/s40780-024-00357-7)
Supplement: Supplementary file 4 — Additional file 4. Patients were divided into four groups according to the number of GDMT and ni-GDMT drugs prescribed at discharge. [file 40780_2024_357_MOESM4_ESM.docx]

Online Resource 4. Patients were divided into four groups according to the number of GDMT and ni-GDMT drugs prescribed at discharge

| Group | Number of GDMT drugs | Number of ni-GDMT drugs |
| --- | --- | --- |
| A | ≥5 | <4 |
| B | <5 | <4 |
| C | ≥5 | ≥4 |
| D | <5 | ≥4 |

GDMT, Guideline-directed medical therapy; ni-GDMT, not included in the Guideline-directed medical therapy
